# CSF tau is associated with impaired cortical plasticity, cognitive decline and astrocyte survival only in APOE4-positive Alzheimer’s disease

**DOI:** 10.1038/s41598-017-14204-3

**Published:** 2017-10-23

**Authors:** Giacomo Koch, Francesco Di Lorenzo, Stefano Loizzo, Caterina Motta, Sara Travaglione, Monica Baiula, Roberto Rimondini, Viviana Ponzo, Sonia Bonnì, Sofia Toniolo, Fabrizio Sallustio, Marco Bozzali, Carlo Caltagirone, Gabriele Campana, Alessandro Martorana

**Affiliations:** 1 Non Invasive Brain Stimulation Unit/Department of Behavioral and Clinical Neurology, Santa Lucia Foundation IRCCS, Rome, Italy; 2 Stroke Unit, Department of Neuroscience, Tor Vergata Policlinic, Rome, Italy; 3 Department of Systems Medicine, University of Rome Tor Vergata, Rome, Italy; 4 Center for Global Health, Italian National Institute of Health, Rome, Italy; 5 Department of Pharmacy and Biotechnology, University of Bologna, Bologna, Italy; 6 Department of Medical and Surgical Sciences, University of Bologna, Bologna, Italy; 7 Neuroimaging Laboratory, Santa Lucia Foundation, IRCCS, Rome, Italy

## Abstract

In Alzheimer’s disease (AD) patients, apopoliprotein (APOE) polymorphism is the main genetic factor associated with more aggressive clinical course. However, the interaction between cerebrospinal fluid (CSF) tau protein levels and APOE genotype has been scarcely investigated. A possible key mechanism invokes the dysfunction of synaptic plasticity. We investigated how CSF tau interacts with APOE genotype in AD patients. We firstly explored whether CSF tau levels and APOE genotype influence disease progression and long-term potentiation (LTP)-like cortical plasticity as measured by transcranial magnetic stimulation (TMS) in AD patients. Then, we incubated normal human astrocytes (NHAs) with CSF collected from sub-groups of AD patients to determine whether APOE genotype and CSF biomarkers influence astrocytes survival. LTP-like cortical plasticity differed between AD patients with apolipoprotein E4 (APOE4) and apolipoprotein E3 (APOE3) genotype. Higher CSF tau levels were associated with more impaired LTP-like cortical plasticity and faster disease progression in AD patients with APOE4 but not APOE3 genotype. Apoptotic activity was higher when cells were incubated with CSF from AD patients with APOE4 and high tau levels. CSF tau is detrimental on cortical plasticity, disease progression and astrocyte survival only when associated with APOE4 genotype. This is relevant for new therapeutic approaches targeting tau.

## Introduction

Alzheimer’s disease (AD) is pathologically characterized by senile plaques, composed of extracellular aggregates of amyloid-β (Aβ) protein, and neurofibrillary tangles formed by hyperphosphorylated tau protein^1^. The accumulation of Aβ begins before the onset of cognitive symptoms and it is followed by the spreading of tau pathology that has been related to the progression of cognitive decline^2–5^. The course of AD is also influenced by genetic factors such as the APOE genotype^6^. Having one or two copies of APOE E4 allele is associated with increased risk of developing AD^7^ and with a more aggressive disease course^8^. Although there is some evidence that APOE4 genotype may be associated with more pronounced tau pathology^9,10^, it is still poorly understood how in AD patients APOE genotype may interact with underlying tau pathology.

A possible *trait d’union* could be the dysfunction of cortical plasticity, which is strictly linked to AD pathology^11^. This is especially relevant since long-term potentiation (LTP) is considered a main neurophysiological correlate of learning and memory^12^. Notably, APOE regulates these mechanisms in an isoform dependent manner^13^ and tau pathology has a detrimental effect on synaptic plasticity^14^. Emerging evidence is promoting a novel framework in which astrocytes are powerful regulators of formation and refinement of synaptic plasticity^15^. Astrocytes are essential for the Aβ-induced tau phosphorylation in neurons^16^ and conversely tau protein plays a central role in inducing astrocytes apoptosis^17^. Astrocytes functions could critically influence brain plasticity and consequently neuronal survival in AD^18^.

Accordingly, we recently identified the disruption of LTP-like cortical plasticity as a core feature of AD by using transcranial magnetic stimulation (TMS)^21^. Indeed, an altered LTP-like cortical plasticity is associated with a more-severe cognitive decline^19^ and with higher CSF tau levels^20^. However, it is still unknown if CSF tau levels interacts with cortical plasticity and with disease severity depending on the APOE genotype. Therefore, in a first series of *in vivo* experiments performed in AD patients, we explored the influence of CSF tau levels on LTP-like cortical plasticity and on clinical progression, according to the APOE genotype. We hypothesized that if APOE4 genotype is associated with more pronounced CSF tau pathology^9,10^, then we would expect in these patients an LTP-like cortical plasticity impairment and a more aggressive disease course, whose might be dependent on the CSF tau levels.

We also aimed at investigating the effects of CSF tau on synaptic impairment by exploring the influence of CSF tau on astrocytes survival^22,23^. Recent works showed that APOE isoforms influence the progression of AD by impairing astrocytes structure and function^24^ and tau protein is thought to play a central role in inducing astrocytes apoptosis^25^. Therefore, in a second series of *in vitro* experiments we incubated normal human astrocytes (NHAs)^26^ with CSF sampled from the same AD enrolled for the TMS experiments in order to verify the effects of CSF tau on astrocytes survival. We hypothesized that higher CSF tau levels would be more detrimental on astrocyte survival especially when associated with APOE4 genotype.

## Results

**Figure 1:**
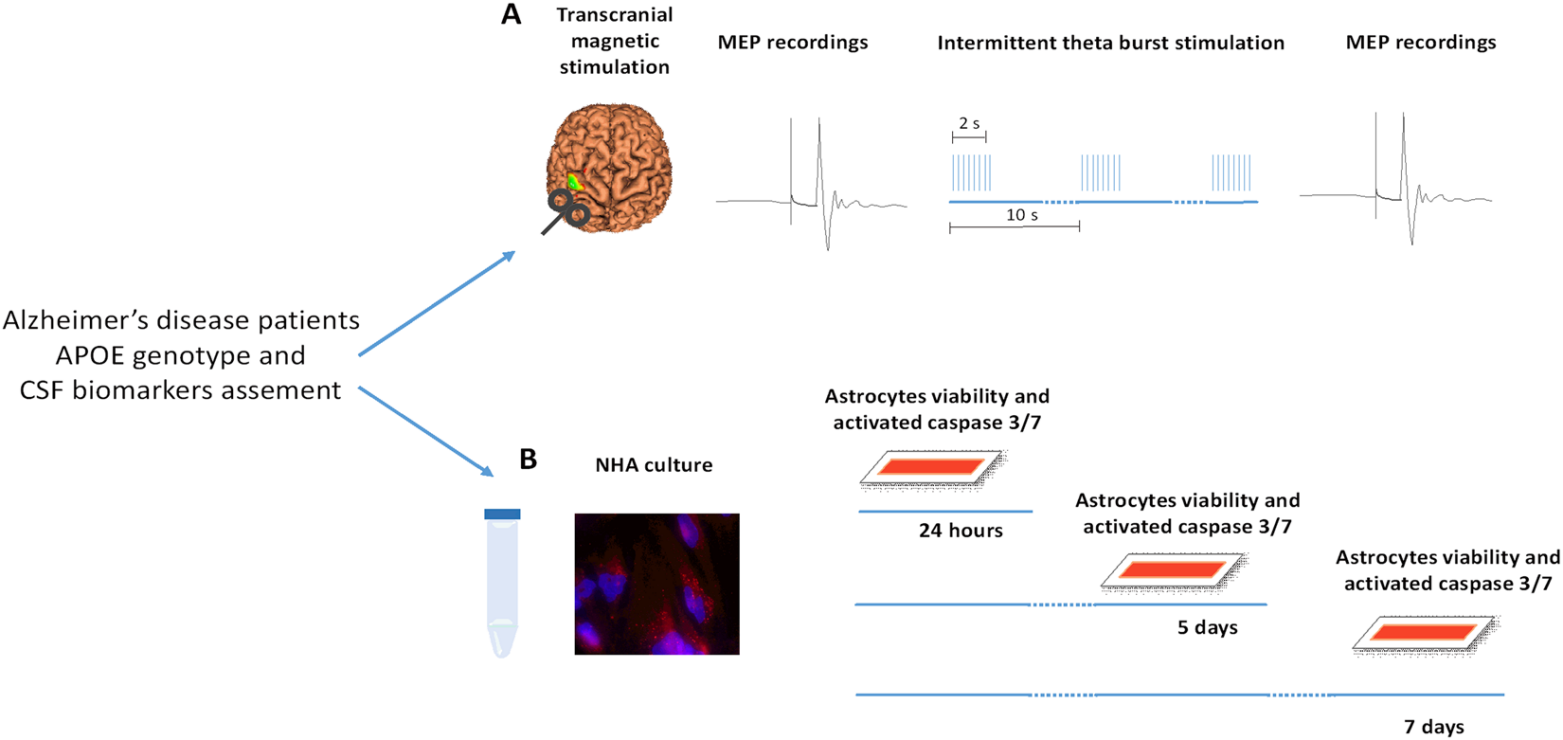
The figure depicts the experimental setting. (**A**) After CSF collection and APOE genotype assessment, all patients underwent TMS applied over the primary motor cortex. MEPs were recorded before and after the iTBS protocol to evaluate cortical plasticity. (**B**) NHAs were incubated in CSF derived from AD patients, divided in groups according to APOE genotype and CSF t-tau levels, and HC. Cell viability and caspase 3/7 levels were evaluated after 24 hours, 5 days and 7 days of incubation.

Forty-one consecutive patients were recruited at the memory clinic of the University Hospital Tor Vergata, admitted for complaining memory symptoms (Fig. 1). APOE4 and APOE3 groups did not differ in gender, education, age at disease onset, disease duration, or Mini-Mental State Examination (MMSE) score at baseline as shown in Table 1. The analysis of CSF protein contents showed increased, although not significant, total tau (t-tau) levels in the APOE4 (n = 20) as compared to the APOE3 (n = 21) group (t-tau: 723.4 ± 369.9 vs 547.2 ± 273.1, p = 0.08). There were no significant differences of Aβ1-42 and p-tau levels between the two groups (Aβ1-42: 317.6 ± 107.0 vs 353.3 ± 145.3, *p* = 0.38; p-tau: 89.7 ± 47.3 vs 80.7 ± 46.0, *p* = 0.54).

**Table 1:** Demographic characterization of Alzheimer’s disease patients and healthy subjects recruited for transcranial magnetic stimulation experiments.

	APOE4	APOE3	HS	*p* value
Subjects (n)	20	21	20	
Sex (M:F)	10:10	11:10	10:10	n.s.
Age at baseline, y (mean ± SD)	70.3 ± 2.4	71.8 ± 2.1	70.1 ± 3.4	n.s.
Formal education, y (mean ± SD)	8.5 ± 4.0	8.1 ± 4.2	8.7 ± 4.4	n.s.
Disease duration, y (mean ± SD)	1.6 ± 0.6	2.1 ± 0.7		n.s.
MMSE at baseline (mean ± SD)	21.7 ± 4.3	23.0 ± 3.4		n.s.
CSF t-tau (pg/ml)	723.4 ± 369.9	547.2 ± 273.1		0.08
CSF p-tau (pg/ml)	89.7 ± 47.3	80.7 ± 46.0		0.54
CSF Aβ1-42 (pg/ml)	317.6 ± 107.0	353.3 ± 145.3		0.38

M: male; F: female; y: years; SD: standard deviation; MMSE: Mini Mental State Examination; n.s.: not significant.

**Figure 2:**
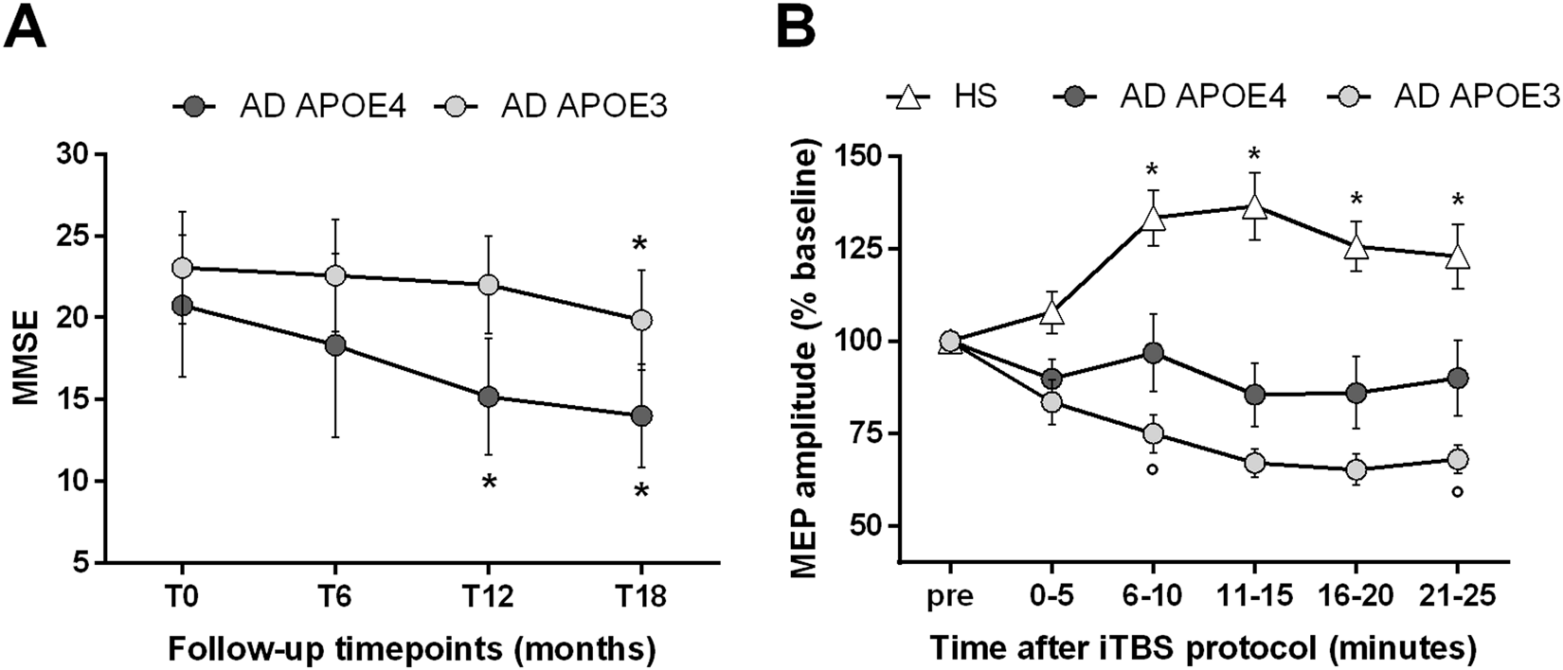
(**A**) Two-way repeated-measures ANOVA performed on MMSE assessed at baseline and at 6, 12 and 18 months in the two groups of AD patients. Error bars indicate standard deviation. *Indicate p < 0.05 in comparison with baseline. (**B**) Two-way repeated-measures ANOVA performed on the iTBS protocol after-effects on MEPs’ amplitude in HS and in the different groups of AD patients according to APOE genotype. Error bars indicate standard error of mean. *Indicate p < 0.05 for the comparison between HS and both APOE3 and APOE4 AD groups. °Indicate p < 0.05 for the comparison between APOE3 and APOE4 group.

During follow up AD patients in both APOE groups were all treated with standard acetylcholinesterase inhibitor (AchEI) therapy (rivastigmine/donepezil). The two groups did not differ in the number of patients undergoing different AchEI treatment (APOE4: 12 patients under rivastigmine and 8 patients under donepezil treatment; APOE3: 13 patients under rivastigmine and 8 patients under donepezil treatment; *p* = 0.75). Follow-up evaluation, with MMSE scores performed at 6, 12 and 18 months, revealed that APOE4 progress faster than APOE3 AD patients as shown by ANOVA (significant GROUP x TIME interaction (F(3, 117) = 3.2782, *p* = 0.024) (Fig. 2A). *Post hoc* analyses revealed that in APOE4 AD patients MMSE scores were lower than baseline evaluation as early as at 12 months (*p* = 0.00003) and showed a further step of decline between the 12 months and the 18 months follow-up (*p* < 0.00001). On the other hand, in APOE3 AD patients MMSE scores became significantly lower respect to baseline only after 18 months (*p* = 0.001).

### Cortical plasticity

The TMS procedures were well tolerated in all subjects. The mean resting motor threshold (RMT) to TMS did not different between APOE3 and APOE4 AD groups, but was lower in both APOE3 (*p* = 0.007) and APOE4 (*p* = 0.001) AD patients compared to healthy subjects (HS) (APOE4: 35.7% ± 1.43 maximum stimulator output; APOE3: 38.6% ± 1.94 maximum stimulator output; HS: 43.7% ± 1.49 maximum stimulator output). Baseline mean motor evoked potentials (MEPs) amplitude did not differ between APOE4, APOE3 AD patients and HS (APOE4: 1.11 ± 0.64 mV; APOE3: 1.06 ± 0.45 mV; HS: 1.09 ± 0.58 mV). ANOVA analysis performed on the intermittent-theta burst stimulation (iTBS) protocol showed a significant GROUP main factor (F(1,39) = 6.08; *p* = 0.01) and GROUP x TIME interaction (F(8, 236) = 2.498; *p* = 0.013), but not for TIME (F(4, 156) = 1.99; *p* = 0.09) main factor. *Post hoc* analyses with Bonferroni correction showed that AD patients with APOE3 and APOE4 genotype had less LTP in comparison to healthy controls at 10, 15, 20 and 25 minutes (*p* < 0.0001 for both comparisons) and that AD patients with APOE3 genotype showed a more pronounced tendency towards long term depression (LTD) in comparison to AD patients with APOE4 genotype at 10 and 25 minutes (both *p* < 0.05) (Fig. 2B **)**.

### Interaction between CSF tau levels, cortical plasticity and cognitive decline according to APOE genotype

**Figure 3:**
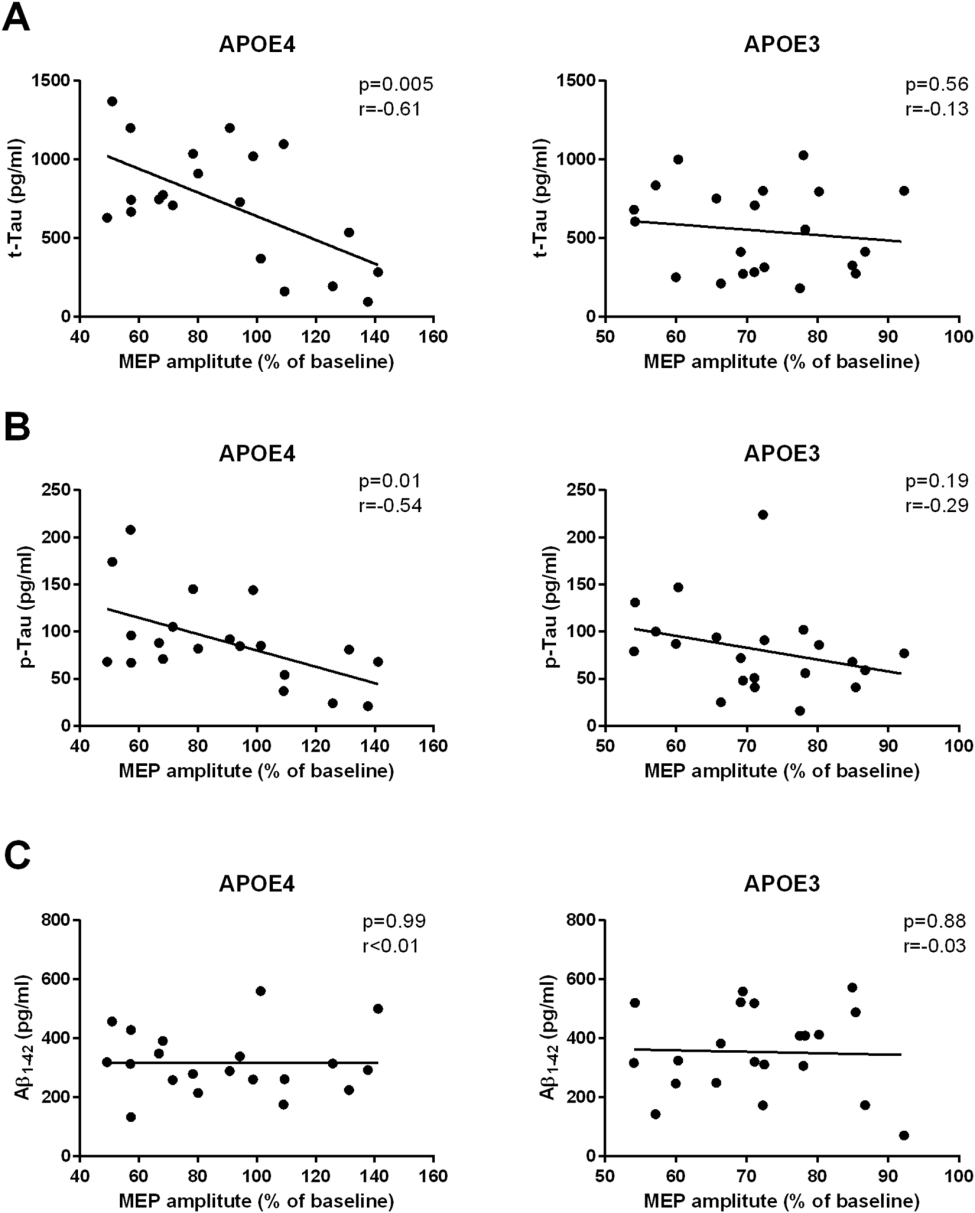
Pearson’s r correlation matrices between the individual amount of mean change in MEPs’ amplitude induced by iTBS protocol and CSF values of t-tau (**A**), p-tau (**B**) and Aβ1-42 (**C**) according to APOE genotype.

We performed separate correlation analyses in order to investigate the relationship between cortical plasticity and CSF biomarkers according to APOE genotype. We found that higher CSF t-tau levels were associated with more impaired cortical plasticity (as indexed by the individual amount of mean change in MEPs’ amplitude after the iTBS protocol) in the APOE4 but not in the APOE3 group (APOE4: *p* = 0.005, r = −0.61; APOE3: *p* = 0.55, r = −0.13) (Fig. 3A). Similar results were found for CSF p-tau (APOE4: *p* = 0.01, r = −0.54; APOE3: *p* = 0.19, r = −0.29) (Fig. 3B), but not for CSF Aβ1-42 levels (APOE4: *p* = 0.99, r = 0.01; APOE3: *p* = 0.88, r = −0.03) (Fig. 3C).

**Figure 4:**
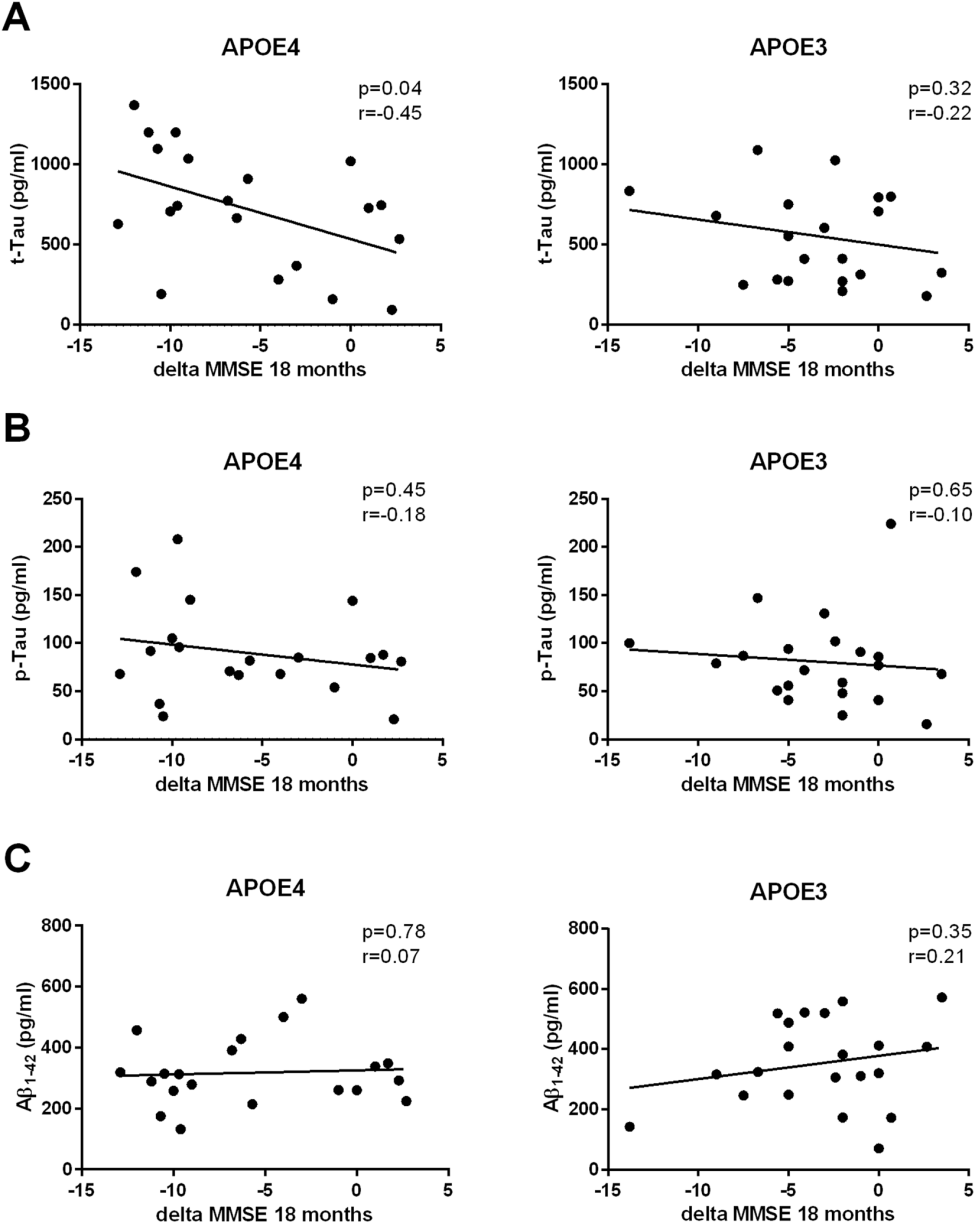
Pearson’s r correlation matrices between cognitive progression, expressed as difference between MMSE score at 18 months follow-up and baseline, and CSF values of t-tau (**A**), p-tau (**B**) and Aβ1-42 (**C**) according to APOE genotype.

We then explored the correlation between CSF biomarkers and cognitive decline. Higher CSF t-tau levels correlated with faster cognitive decline (as indexed by delta MMSE score) only in the APOE4 but not in the APOE3 group (APOE4: *p* = 0.04, r = −0.45; APOE3: *p* = 0.33, r = −0.22) (Fig. 4A). On the other hand, CSF levels of p-tau (APOE4: *p* = 0.45, r = −0.18; APOE3: *p* = 0.65, r = −0.10) (Fig. 4B) and Aβ1-42 did not correlate with cognitive decline in both APOE groups (APOE4: *p* = 0.78, r = 0.07; APOE3: *p* = 0.35, r = −0.21) (Fig. 4C).

**Figure 5:**
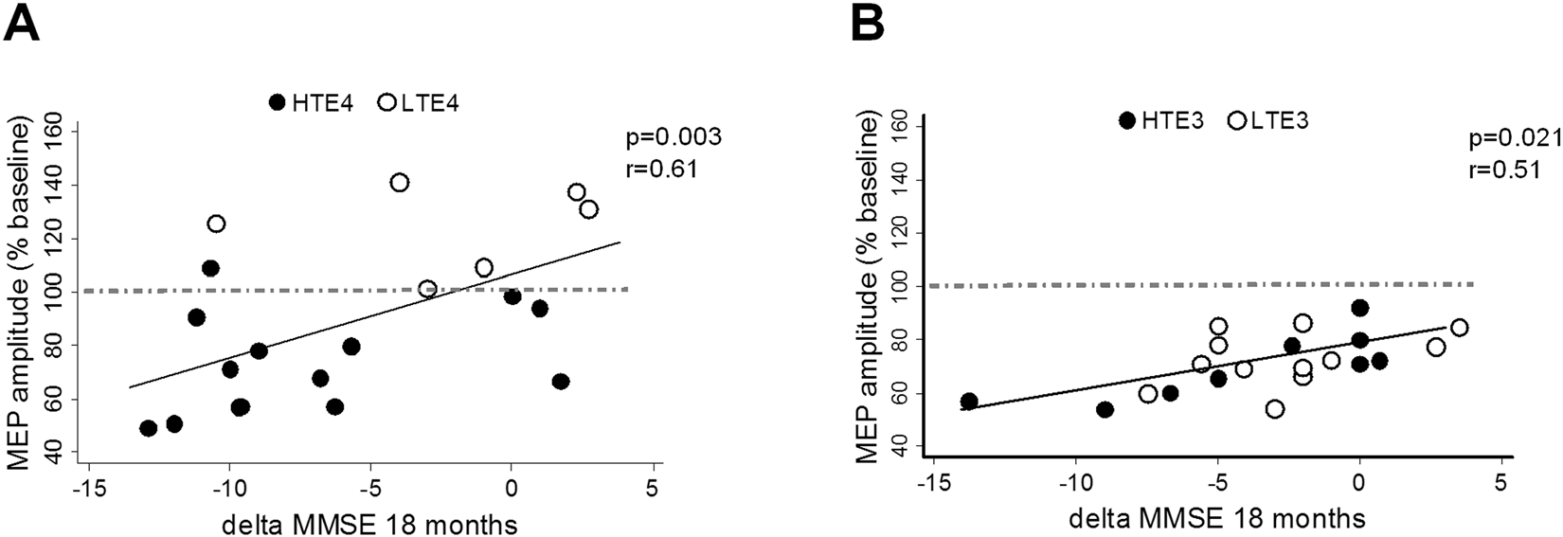
Pearson’s r correlation matrices between the individual amount of mean change MEPs’ amplitude induced by iTBS and the cognitive progression, expressed as delta of MMSE scores at 18 months follow up, in APOE4 (**A**) and APOE3 (**B**) AD. White dots represent low (<680 pg/mL) CSF t-tau levels, black dots represent high (>680 pg/mL) CSF t-tau levels.

We also found that AD presenting with more altered cortical plasticity showed a more pronounced cognitive decline at 18 months, in agreement with our previous findings^19^. In fact, the mean individual response to the iTBS correlated with delta MMSE scores at 18 months in both APOE3 and APOE4 AD patients (APOE3: *p* = 0.003, r = 0.61; APOE4: *p* = 0.021, r = 0.51). However, CSF t-tau levels had a strong influence on the relationship between cortical plasticity and cognitive decline depending on the APOE genotype. In this analysis, AD patients were stratified according to CSF t-tau levels into high CSF t-tau levels and low CSF t-tau levels, using as cut-off value the median of CSF tau levels of our entire sample (680 pg/ml). We found that APOE4 patients with high CSF t-tau levels showed a response to the iTBS protocol towards LTD, while conversely, APOE4 patients with low CSF t-tau showed a tendency to form LTP (Fig. 5A). On the other hand, in the APOE3 group, we did not observe any specific distribution according to CSF t-tau levels (Fig. 5B), since all patients showed a uniform response to the iTBS protocol towards LTD.

### Normal human astrocytes cultures

A second series of combined *in vitro* experiments was performed on cell cultures using human CSF as incubation medium as previously reported^27^. NHAs cultures were incubated with CSF collected from AD patients recruited from those enrolled for the assessment of cortical plasticity. CSF samples from 16 AD patients grouped according to CSF t-tau levels, using as cut-off value the median of CSF tau levels of all our sample (680 pg/ml), and APOE genotype, resulting in 4 groups (each one consisting of four subjects), namely APOE3 low t-tau levels (LTE3), APOE3 high t-tau levels (HTE3), APOE4 low t-tau levels (LTE4) and APOE4 high t-tau levels (HTE4). The four resulting groups were matched for sex, age, MMSE at the baseline and disease onset (Table 2).

**Table 2:** Demographic and clinical characterization of Alzheimer’s disease patients and control subjects recruited for experiments on normal human astrocytes incubated with CSF.

	LTE3	LTE4	HTE3	HTE4	CTRL	*p* value
Subjects (n)	4	4	4	4	4	
Sex (M:F)	2:2	2:2	2:2	2:2	2:2	n.s.
Age at baseline, y (mean ± SD)	74.2 ± 2.2	74.3 ± 1.9	72.3 ± 1.9	71.4 ± 2.8	69.1 ± 3.8	n.s.
Disease duration, y (mean ± SD)	1.9 ± 0.5	1.8 ± 0.6	2.2 ± 0.6	1.7 ± 0.8	n.a.	n.s.
MMSE at baseline (mean ± SD)	23.5 ± 2.8	23.1 ± 2.7	21.3 ± 2.9	21.4 ± 2.9	n.a.	n.s.
CSF t-tau (pg/ml)	386.5 ± 51.6^a^	378.3 ± 49.6^a^	1008.0 ± 199.4	1010.2 ± 180.7	178.3 ± 115.5^a^	<0.01
CSF p-tau (pg/ml)	49.5 ± 18.0^a^	61.4 ± 22.5^a^	128.6 ± 33.8	110.2 ± 30.7	28.3 ± 9.5^a^	<0.01
CSF Aβ1-42 (pg/ml)	318.7 ± 34.1	280.4 ± 39.6	360.2 ± 40.2	314.9 ± 27.8	798.5 ± 88.7^b^	<0.01

M: male; F: female; y: years; SD: standard deviation; MMSE: Mini Mental State Examination; n.s.: not significant. *p* value expressed for Kruskal-Wallis/

^a^Difference vs HTE3 and HTE4.

^b^Difference vs CTRL.

### Astrocytes viability

**Figure 6:**
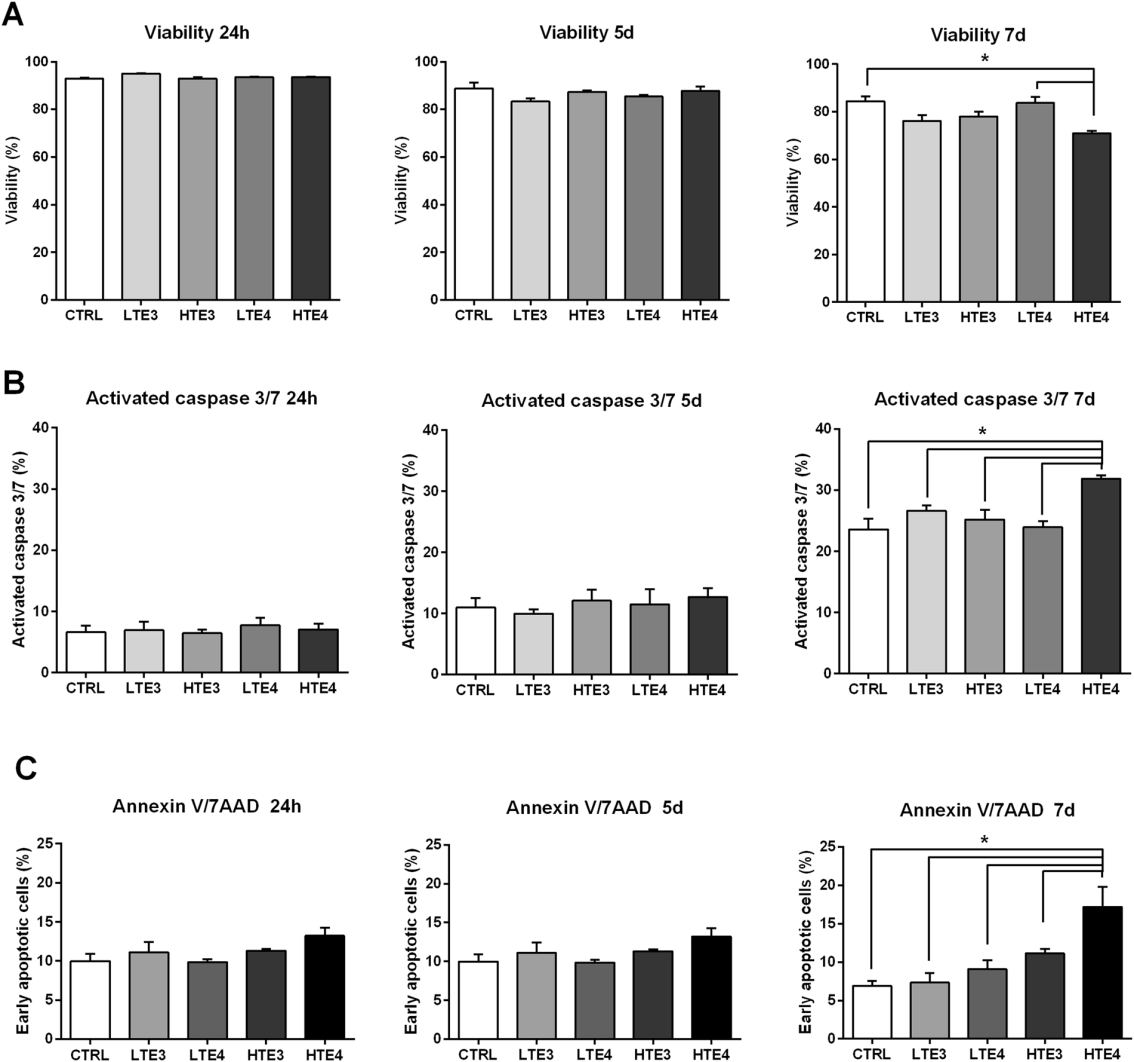
One-way ANOVA on NHAs viability (**A**) caspase 3/7 levels (**B**) and early apoptotic levels (**C**) at 24 hours, 5 days and 7 days of incubation with CSF of AD patients, divided in groups according to APOE genotype and t-tau levels, and control subjects. Results are expressed as mean ± standard deviation of three independent experiments carried out in duplicate. *Indicate p < 0.05 for the post hoc multiple comparisons test.

To assess the effect of CSF incubation on physiological status of astrocytes, we analyzed cell viability using 7-amino actinomycin D (7-AAD), a membrane impermeant dye that is generally excluded from viable cells. A general time-dependant decrease in NHAs viability was detected during long-term CSF incubation. We found no differences among all CSF treated samples at 24 hours and 5 days **(**Fig. 6A **)**; conversely, at 7 days HTE4 sample showed significantly lower viability when compared to LTE4 and control samples (Kruskal-Wallis test: *p* = 0.0002; Dunn’s multiple comparisons post hoc test: HTE4 vsHC*p* = 0.03; HTE4 vs LTE4 *p* = 0.04). Hence, the results of this experiment show that the coexistence of high CSF levels of t-tau and APOE4 genotype is able to influence astrocytes viability; conversely CSF t-tau levels do not induce any change when associated to APOE3 genotype.

### Levels of apoptosis in NHA

Cell viability loss is often linked to apoptosis and the activation of caspases, a milestone in the path of programmed cell death, leads to proteolytic cleavage of several neuronal proteins in AD^28^. Several studies suggest that activation of caspases and cleavage of cellular proteins may contribute to astrocyte injury and damage in the AD patient brain^29^. Thus, to evaluate the effect of CSF incubation on apoptosis in NHAs, we employed two approaches to detect early apoptosis by measuring the activity of caspase 3/7 and annexin V/7AAD after 24 hours, 5 days and 7 days of incubation. We found that activated caspase 3/7 levels were similar among groups until day 7 **(**Fig. 6B **)**, when we observed higher activated caspase level in the HTE4 respect to LTE4, APOE3 and control groups (ANOVA *p* < 0.0001; HTE4 vs all *p* < 0.01). Similar results were obtained from Annexin V assay **(**Fig. 6C **)**. In fact, after 24 hours and 5 days of incubation we did not observe any variation in apoptotic levels while astrocytes treated with HTE4 for 7 days showed the highest percentage of early apoptotic cells, if compared to all other groups (ANOVA p < 0.0001; HTE4 vs all p < 0.01). No differences were observed between LTE3 and HTE3 **(**Fig. 6B and C **)**, suggesting that t-tau levels might be the factor responsible for cell apoptosis only in NHAs treated with CSF from patients with APOE4 isoform.

## Discussion

We provide novel evidence that the presence of APOE polymorphisms imply different mechanisms of CSF tau-related dysfunction in AD patients. Our work reveals that high CSF tau levels are associated with impaired cortical plasticity and disease progression only in AD patients carrying APOE4 but not APOE3 genotype. In parallel, we also found that CSF tau levels influence apoptosis in NHAs when incubated with CSF collected from AD patients with APOE4 but not APOE3 genotype. Taken together these findings reveal that CSF tau levels are linked to cortical plasticity, cognitive decline and astrocytes survival only when associated with APOE4 genotype.

In our study, APOE3 and APOE4 AD patients did not initially differ in terms of neuropsychological symptoms and degree of functional status, thus making them indistinguishable from a clinical point of view, at least at the time of their first visit to the memory clinic. Indeed, our follow up evaluations revealed that the two groups show divergent clinical progression, since APOE4 group worsen faster than APOE3, accordingly to previous evidence^30–32^.

Both APOE4 and APOE3 AD patients showed, as expected, an impairment of LTP-like cortical plasticity^19,20^. Interestingly, APOE3 patients showed an evident tendency to form paradoxical LTD instead of the expected LTP-like cortical plasticity in comparison to APOE4 patients. This different profile of LTP-like cortical plasticity dysfunction can be explained according to the extensive experimental works in animal models of AD revealing that APOE polymorphism has a profound influence on disease-related synaptic dysfunction^15^. These studies showed that APOE selectively impairs glutamatergic neurotransmission and LTP by reducing NMDA and AMPA receptor functions^33^. In particular it was found that LTP is more impaired in APOE2 and APOE3 in comparison to APOE4 mice^34,35^. This is compatible with our findings, showing a relative less impaired LTP in APOE4 in comparison with APOE3 AD patients. It is possible that such differences could involve complex interactions with glutamatergic transmission^36^, although the exact mechanisms remain to be clarified in further studies.

In our sample, faster cognitive decline was associated with more impaired LTP-like cortical plasticity, a condition observed in both APOE3 and APOE4 group, confirming previous observations that an altered cortical plasticity could be involved in fastened clinical worsening^19,20,22^. However, we found a striking difference when considering the interaction between disease progression and CSF tau levels according to the APOE genotype. Only in APOE4 AD patients there is a strong association between cognitive decline, impaired cortical plasticity and CSF t-tau values, suggesting a relative susceptibility to APOE4 patients to the amount of tau-related pathological burden. On the other hand, this phenomenon is completely absent in APOE3 AD patients, which are indeed characterized by a strong correlation between LTP-like cortical plasticity dysfunction and cognitive decline, which is totally independent from CSF tau levels. Therefore, our data show a clear dependency on underlying CSF tau levels that is exclusive for APOE4 genotype. This finding is clinically relevant especially in light of recent findings showing that AD patients with very high levels of CSF tau and phosphorylated tau at threonine 181 (p-tau^181^) exhibit worse clinical outcomes over time, including faster progression of cognitive deficits and a higher mortality^5^. In addition, they are also compatible with a recent work showing that CSF tau and p-tau^181^ levels, but not Aβ42 levels, predict subsequent rates of whole-brain and regional atrophy in AD over the next three years^37^. Other recent clinical evidence also supports this result: for instance, a recent longitudinal study of CSF biomarkers found that APOE4 genotype influences dramatically markers of neuronal injury inducing a constant increase of CSF t-tau and p-tau181 levels^36^, and higher CSF tau levels were specifically correlated with increased tau in the temporal lobe, a region involved in memory processing^38^. Indeed, there is an increased risk of progression from mild cognitive impairment to AD with elevated CSF t-tau and p-tau and with the presence of the APOE4 genotype, but not with decreased CSF Aβ42 levels^39^. Moreover, APOE4 may accelerate onset of dementia and neuronal degeneration by differentially impairing the maintenance of synaptic strength and reducing glutamate signaling proteins, even in the absence of overexpression of APP and Aβ APOE4^39^.

The current neurophysiological and clinical findings were also paralleled by similar interactions between CSF tau levels and APOE genotype when examining astrocytes vulnerability. Notably, the CSF samples used as incubation medium for the astrocytes cultures were collected from the same AD patients in whom we assessed cortical plasticity. We found that NHAs were less viable and more susceptible to apoptosis when incubated with CSF obtained from APOE4 patients with high levels of CSF tau but not from APOE3 ones. We also found a similar phenomenon when evaluating the activation of caspases, a milestone in the path programmed of cell death known as apoptosis, occurring in AD and leads to proteolytic cleavage of several neuronal proteins^40^. In particular, the caspase-3 inhibits the production of ROS and is necessary for the efficient execution of apoptosis, while it is necessary for the effector caspase-7 cell detachment by apoptosis^41^. Several studies suggest that the plaques, tangles, and the activation of caspases share a common path and that induced caspase cleavage of tau plays an important role in the development of the disease neurofibrillary tangle^42^. Since caspase-3 activity can be also induced independently of apoptosis^43^ and apoptosis is not necessarily caspase-dependent^44^, we performed also the annexin V/7AAD assay in order to confirm the data on apoptosis deriving from caspase 3/7 activity. In fact, annexin V is a 35–36 kDa Ca2+-dependent phospholipid-binding protein with high affinity for membrane phosphatidylserine (PS) that binds to PS exposed on cell surface of apoptotic cells^45^.

This is relevant since a growing body of evidence shows that alterations in astrocytic functions have potentially catastrophic consequences for neurons^46,50^. Moroever astrocytes apoptosis has been reported in several experimental^25,47^ and clinical neuropathological conditions including AD^48,49^ thereby reinforcing the notion that damage of astrocytes can contribute to disease progression.

Regarding the possible mechanisms underlying the current findings we can only speculate about possible sites of interaction between CSF tau and APOE. One possibility is that CSF tau combines with the APOE receptors in the extracellular space, indirectly modulating the intracellular transduction signaling pathways that regulate tau kinases activity^51^. In alternative, APOE4 and its C-terminal-truncated fragments could regulate directly tau phosphorylation and the formation of neurofibrillary tangle-like inclusions in the intracellular space of neurons^52^. At this regard, studies investigating the relationship between apoE and the microtubule-stabilizing protein tau showed that APOE3 binds tightly to tau, whereas APOE4 does not. These data provided the basis for hypothesizing a role for APOE in microtubule stabilization. While binding of APOE3 to tau is proposed to stabilize microtubules and inhibit its hyperphosphorylation, binding deficiency of APOE4 would allow for tau self-association, hyperphosphorylation, and subsequent formation of paired helical filaments, the building blocks of neurofibrillary tangles in AD^53^. This experimental evidence could account for our current findings. In fact, while CSF collected from APOE3 AD patients with different tau values did not influenced astrocytes survival, the samples collected from AD APOE4 patients affected astrocytes viability and apoptosis depending on the CSF tau levels. Therefore, our data show that only APOE4 may influence astrocyte survival and functionality depending on CSF tau levels. Given the established role of astrocytes in controlling the complex synaptic machinery at the basis of the mechanisms of LTP, we propose that the interaction between altered synaptic plasticity, CSF tau levels and APOE genotype could explain why AD patients with APOE4 allele tend to have more impaired cortical plasticity and eventually a more aggressive course of disease. On the other hand, the different pathophysiological mechanisms linked to the presence of APOE3 allele remain to be elucidated.

A limitation of the current study is that we cannot exclude that other interactions could have taken place involving other molecules apart from tau in the experiments with astrocytes cultures. Therefore, further *in vitro* experiments are needed to better identify the interaction between Aβ42, t-tau and p-tau and astrocytes survival, to define the metabolic pathways involved in these mechanisms. Moreover additional work is required to better establish the pathophysiological role of CSF tau oligomers in relation to APOE polymorphism.

In conclusion, here we used a novel translational model to study the mechanisms of cortical synaptic plasticity impairment occurring in AD. Our findings suggest that: i) CSF tau drives cortical plasticity impairment and cognitive decline only in the presence of APOE4 genotype, while APOE3 patients seem to be characterized by other pathological mechanisms; ii) APOE4 patients CSF tau levels can influence astrocytes survival, independently from Aβ levels. Thus, our data point to the existence of independent pathophysiological pathways mediating cognitive decline in AD patients according to the APOE polymorphism. These findings could be relevant to specifically address new diagnostic and therapeutic approaches targeting tau, in order to select AD patients according to specific clinical and biological characteristics.

## Methods

### Subjects

Forty-one consecutive patients recruited at the memory clinic of the University Hospital Tor Vergata, admitted for complaining symptoms. After the first visit to our Centre, all patients underwent for diagnostic purposes a complete clinical investigation in a period not superior to 60 days, including medical history, neurological examination, Mini-Mental State Examination (MMSE), a complete blood screening, neuropsychological assessment, magnetic resonance or CT imaging, and lumbar puncture for CSF analysis (Table 1 **)**. Patients fulfilled the clinical criteria of dementia as defined by the DSM-IV and probable or possible AD according to the criteria of the National Institute of Neurological and Communicative Disorders and Stroke and the Alzheimer’s disease and Related Disorders Association^54^. Disease duration was calculated using standardized semi structured questionnaire^55^. Neurophysiological examinations were performed at the Santa Lucia Foundation within 30 days from CSF sampling. Patients included in this study did not receive drugs that could have modulated cerebral cortex excitability such as Acetylcholinesterase inhibitors (AchEI), L-Dopa or dopamine-agonists antidepressants or any other neuroactive drugs (i.e. benzodiazepines, anti-epileptic drugs or neuroleptics) in the 90 days prior to TMS evaluation. Exclusion criteria were the following: patients with isolated cognitive deficits, patients with clinically manifest acute stroke in the last 6 months showing a Hachinsky scale score >4, and a radiological evidence of ischemic lesions, Aβ1-42 CSF values >600 pg/mL. After the neurophysiological assessment patients started treatment with rivastigmine patch (n = 26) or donepezil (n = 15) and were followed longitudinally with clinical assessments and MMSE at 6, 12 and 18 months. Twenty age, sex- and education-matched healthy subjects (HS) were recruited as controls. All participants or their legal guardian provided written informed consent after receiving an extensive description of the study. The study was performed according to the Declaration of Helsinki. The ethics committee of the Santa Lucia Foundation approved this protocol (Prot. CE/AG4/PROG.392-08).

### CSF analysis and APOE genotype

CSF analysis was performed within routine clinical testing in patients with cognitive impairment. The CSF samples were collected in a polypropylene tube, directly transported to the local laboratory for centrifugation at 2000 × g at +4 °C for 10 minutes to eliminate cells and cellular debris, then stored at −80°. CSF t-tau and p-tau phosphorylated at Thr181 concentrations were determined using a sandwich ELISA (InnotesthTAU-Ag, Innogenetics, Gent, Belgium). Aβ1-42 levels were determined using a sandwich ELISA (Innotest® ß- amyloid (1–42), Innogenetics, Gent, Belgium), specifically constructed to measure Aβ-amyloid containing both the first and 42nd amino-acid, as previously described^56^. APOE genotyping was performed using a standard PCR. Two groups of AD patients were classified using the APOE genotype. APOE4 group consisted of fifteen E3/E4 and five E4/E4 allele carriers AD patients while APOE3 group consisted of twenty-one AD patients homozygous for E3 allele (Table 1).

### Transcranial magnetic stimulation

In a first series of *in vivo* experiments we investigated the role of APOE genotype on cortical plasticity by applying theta burst stimulation (TBS) protocols^29^ in a large sample of AD patients. TBS, a form of repetitive TMS has been put forward as a reliable tool to examine cortical plasticity in AD patients in analogy with hippocampal plasticity assessed in animal models of AD. We verified how APOE polymorphism eventually affected LTP-like cortical plasticity by interacting with CSF levels of total tau, p-tau181 and Aβ42. Motor evoked potentials (MEPs) were recorded from the right first dorsal interosseous muscle using 9 mm diameter, Ag–AgCl surface cup electrodes. A monophasic Magstim 200 device (Magstim Co, UK) was used to define the motor hot spot and to assess MEPs size using standard 70 mm figure-of-eight shaped coil. The motor hot-spot was defined as the location where TMS consistently produced the largest MEP size at 120% of resting motor threshold (RMT) in the target muscle^57^. A second coil was connected to a biphasic Super Rapid Magstim stimulator (Magstim Co, UK) to deliver TBS. In the intermittent-TBS (iTBS) protocol, a 2 s train of TBS was repeated twenty times, every 10 seconds for a total of 190 seconds (six-hundred pulses)^58^. The intensity of the test pulse was adjusted so that it evoked a MEP of about 1 mV peak-to-peak amplitude in each individual. Twenty MEPs were collected and averaged at baseline. Then, over the same hot-spot, twenty MEPs were recorded at 1–5, 6–10, 11–15, 16–20 and 21–25 minutes after TBS and averaged.

### Primary astrocytes culture

Lonza NHAs were purchased from Euroclone (Pero, Italy) and cultured according instructions. These experiments were performed at the University of Bologna. NHAs were seeded in 75 cm^2^ flasks containing astrocyte basal medium (ABM™) at 37 °C in 5%CO2 humidified incubator, changing the growth medium the day after and every other day thereafter. The cells were sub-cultured when they were 70–80% confluent and containing many mitotic figures. New culture flasks were prepared by adding pre-warmed medium and the flasks were returned in the incubator. In a sterile field the medium was aspirated from flask and the cells were rinsed with 5 ml of room temperature HEPES-BSS and after a brief swirling were removed and replaced with 2 ml of trypsin/EDTA solution and placed the flask in the 37 °C incubator for 3 to 4 minutes for trypsinization. After detached, the cells suspension was quickly transferred to a sterile 15 ml centrifuge tube. The cell suspension was then centrifuged at 160 × g to 200 × g for 5 minutes at 2 °C to 8 °C. The supernatant was aspirated and the cell pellet was re-suspended in 2 ml of AGM™ medium (Lonza) and the cells were seeded at density of 5,000 cells/cm2 to a tissue culture flask or dish (pre-filled with pre-warmed medium).

The cells were seeded into a 24 cell plates at the recommended density (10,000 cells/cm^2^). The day after medium was changed with 500 µl of fresh medium and 100 µl of CSF from AD patients with different tau levels was added to a final 1:6 dilution^38^. Using the same methods, cells were also incubated in CSF derived from healthy control (HC) subjects. In accordance with the institution’s ethical standards which prevent the recruitment of healthy people for CSF collection, the control group is composed of astrocytes treated with CSF collected from patients that received lumbar puncture and did not show any feature of neurological disorder.

Cell viability was assessed by Guava ViaCount Reagent (Millipore) that contains 7-amino-actinomycin D (7-AAD). Briefly, NHA (10^5^ cells/sample) were diluted 1:1 with the reagent and incubated at room temperature in the dark for 5 min before detection with Guava EasyCyte 5 Flow Cytometer (Millipore); at least 10,000 cells/sample were analyzed.

Apoptosis was also assayed by measuring caspase-3/7 activity using Guava Caspase 3/7 FAM kit (Millipore) following manufacturer’s instructions. NHA (10^5^ cells/sample) were harvested following 24 h, 5 and 7 days treatment with AD patients’ CSF, and incubated in Caspase Reagent Working Solution for 1 hour at 37 °C in a CO_2_ incubator. Then cells were washed twice and labeled with Caspase 7-ADD working solution for 10 min at room temperature. Astrocytes were analyzed on a Guava EasyCyte 5 flow cytometer (Millipore); at least 10,000 cells/sample were analyzed.

To confirm data on NHA apoptosis obtained with Caspase 3/7 assay, phycoerythrin-conjugated annexin V (annexin V-PE) and 7-AAD (Guava Nexin Reagent, Millipore) method was used to determine the percentage of viable, early-apoptotic and late apoptotic/necrotic cells by flow cytometry. Astrocytes (10^5^ cells/sample) were harvested following 24 h, 5 and 7 days treatment with AD patients’ CSF and resuspended in 100 μl of complete medium; the cells were then stained with 100 μl annexin V-PE and 7-AAD for 20 min at room temperature in the dark, following manufacturer’s instructions, and analyzed on a Guava EasyCyte 5 flow cytometer (Millipore). Three populations of cells can be distinguished by this assay: viable cells (annexin V-PE and 7-AAD negative), early apoptotic cells (annexin V-PE positive and 7-AAD negative), and late stages apoptosis or necrotic cells (annexin V-PE and 7-AAD positive).

### Statistical analysis

The analysis was performed using SPSS for Windows version 11.0. The assumptions and calculations at the bases of the sample size were 90% power to detect, using a two-sided alpha-level of 5%, a difference between APOE groups of at least 2.2 point in MMSE at 18 months. Data were presented as mean ± standard deviation (SD) or as mean ± standard deviation (SEM) as reported. Kruskal-Wallis analysis of variance was performed for analyzing the difference among 3 or more independent groups, followed by Mann-Whitney U test with Bonferroni correction when significant, χ^2^ test was used for categorical variables. Univariate associations between iTBS-induced cortical plasticity (individual mean value), cognitive decline (delta score with baseline evaluation) and CSF biomarkers levels were investigated using Pearson’s correlation analysis or Spearman’s Rho analysis, as appropriate. For TMS experiments, two-way repeated measure ANOVAs were performed on MEPs amplitude expressed as percentage of change in comparison to baseline for iTBS protocol with TIME (1–5, 6–10, 11–15,16–20, and 21–25 min after iTBS) as within subjects factors and GROUP (APOE3, APOE4 and HS) as between subjects factor. The Greenhouse-Geisser correction was used for non-spherical data. When a significant main effect was reached, paired t-test with Bonferroni correction was employed. A *p* value of <0.05 was considered to be significant.

For *in vitro* assays, every experimental group was composed by cells treated with CSF from four different patients. In viability and apoptosis measurements performed with Guava, each reading at least 10.000 cells/sample was analyzed in triplicate. The statistical analysis was performed using GraphPad Prism. A one-way ANOVA followed by a *post-hoc*Bonferroni multiple comparison test was used for parametric data; a Kruskal-Wallis test, followed by Dunn’s multiple comparisons test was used for non-parametric data. Values of *p* < 0.05 were considered as being significant.
